# Special Issue: Molecular Research in Asthma and Allergy

**DOI:** 10.3390/ijms27219822

**Published:** 2026-11-02

**Authors:** Sylwia Smolinska-Wilczynska

**Affiliations:** Department of Clinical Immunology, Faculty of Medicine, Wroclaw Medical University, Parkowa 34, 51-616 Wroclaw, Poland; sylwia.smolinska@umw.edu.pl

## 1. The Molecular Landscape of Allergic Disease

Asthma and allergic diseases arise from the interplay of epithelial barriers, innate and adaptive immunity, inherited susceptibility, and environmental exposure. Their clinical manifestations are diverse, but shared inflammatory circuits link disease processes in the airways, skin, and other organs. The traditional view of allergy as a predominantly immunoglobulin E (IgE)- and T-helper 2-driven reaction is therefore necessary but incomplete. Type 2 cytokines and epithelial alarmins remain central to many clinical phenotypes, while regulatory immune populations, tissue-specific responses, and non-type 2 pathways further shape disease heterogeneity [1,2,3].

This Special Issue, “Molecular Research in Asthma and Allergy”, brings together ten contributions published in 2025–2026. Their combined scope spans experimental immunology, pediatric clinical observations, environmental and metabolic modifiers, allergen passage through skin, molecular diagnostics, proteomics, and targeted therapy.

## 2. Immune-Cell Plasticity and Tissue-Specific Inflammatory Networks

The contributions by Grän and colleagues, and Lipińska-Opałka and colleagues place B-cell biology at the center of the discussion. In experimental contact hypersensitivity, B-cell-specific deletion of NFATc1 was associated with attenuated inflammatory responses and an increased regulatory IL-10-producing B-cell compartment. The pediatric clinical study, in turn, described changes in circulating B-cell subsets in children with allergic diseases. Although the models, populations, and endpoints differ, they invite the same question: how do distinct B-cell phenotypes balance inflammatory amplification against immune restraint? The answer will require functional validation rather than reliance on surface-marker frequencies alone.

A second group of papers considers cytokine activity at the skin–immune interface. Łacwik and colleagues reviewed the IL-31/IL-33 axis in atopic dermatitis, including links among epithelial signaling, pruritus, and neuroimmune feedback. Zaryczańska and colleagues examined epidermal IL19 and IL37 transcript patterns in affected and apparently non-lesional skin. Together, these contributions show why tissue context matters: visible lesion boundaries may not coincide with molecular inflammatory boundaries, and a change in cytokine abundance is not automatically evidence of a causal therapeutic target. More broadly, emerging understanding of alarmin signaling indicates that epithelial tissues actively instruct, rather than merely undergo, immune responses [2].

## 3. Susceptibility Is Shaped by Exposure, Metabolism, and Barrier Access

The question of why allergic disease develops in one individual but not another cannot be addressed solely by measuring inflammatory mediators. Dębińska and colleagues examined genetic variation in TNS1 and NRXN1 alongside early-life tobacco-smoke exposure in relation to an asthma–eczema complex phenotype, illustrating the importance of evaluating susceptibility in its environmental context. Ekpruke and colleagues, using a house-dust-mite model, investigated ketogenic dietary modulation of airway inflammation and reported sex-dependent immunological effects. These findings should not be interpreted as evidence for a clinical dietary intervention in asthma; they instead generate testable hypotheses about immunometabolism, biological sex, and exposure-dependent inflammatory trajectories.

Barrier access forms another crucial step between exposure and sensitization. Kiani and colleagues quantified the penetration of wheat-derived proteins into epidermal layers after epicutaneous application, addressing a mechanistic prerequisite for cutaneous allergen encounters. Their work belongs within a broader body of research emphasizing the skin barrier as a potential route of food-allergen sensitization [4]. Importantly, demonstration of epidermal penetration is not synonymous with proof of subsequent sensitization or clinical allergy. These distinctions are essential for translating molecular observations into preventive strategies.

## 4. Toward Biomarkers That Inform Clinical Decisions

Precision allergy medicine requires more than increasingly detailed molecular measurement. Gheorghita and colleagues reviewed component-resolved IgE assays for grass-pollen allergy, emphasizing their ability to separate patterns of genuine sensitization from cross-reactivity and to refine interpretation in appropriate clinical contexts. Meanwhile, Rzetecka-Mańka and colleagues explored proteomic differences among young children with asthma and IgE-associated allergy. The two approaches represent complementary levels of resolution: allergen-component recognition can assist a defined diagnostic question, whereas unbiased proteomic profiling may reveal candidate signatures not captured by conventional tests.

The distinction between analytical novelty and clinical utility deserves emphasis. IgE sensitization does not by itself demonstrate symptomatic allergy; candidate proteomic markers likewise require replication, prospective testing, and assessment of added value over established clinical indicators. For heterogeneous asthma, the move toward molecular endotypes and therapy-linked biomarkers is an ongoing development rather than a completed transition [1,3].

## 5. Targeted Intervention and the Limits of Pathway-Specific Thinking

Biologics targeting type 2 mediators have transformed the management of selected patients with severe allergic inflammation. Yet, suppressing one pathway may expose or alter activity in others. Tsai and Yu reviewed inflammatory phenotype switching during biologic treatment in pediatric skin disease, drawing attention to psoriasiform changes and other shifts in immunological presentation. This contribution makes a broader point relevant to asthma and allergy: a treatment response is not a fixed property of a single baseline biomarker but a dynamic interaction between therapeutic pressure and interconnected immune circuits. Advances in targeting epithelial alarmins further reinforce the need to monitor both expected benefits and broader immune consequences [2,5].

## 6. Priorities for the Next Phase of Research

Viewed together, the ten papers suggest four priorities. First, mechanistic studies should connect immune-cell phenotype with demonstrated function, particularly when proposing regulatory populations or cytokine transcripts as biomarkers. Second, studies of susceptibility should incorporate exposure timing, sex, age, tissue integrity, and genetic background wherever feasible. Third, exploratory multi-omics results need external validation and explicit evidence that they improve diagnosis, prognosis, or treatment selection. Finally, targeted-treatment studies should account for immune-network plasticity, including phenotype switching and outcomes beyond the initially inhibited pathway.

These priorities are especially important in pediatric disease, when immunological development and the timing of exposures may shape long-term trajectories. Translational designs that combine longitudinal cohorts, mechanistic assays, and clinically interpretable endpoints will be better positioned to discriminate initiating events from markers of established inflammation. A molecular finding becomes valuable to patients only when its robustness, clinical relevance, and limitations are defined.

## 7. Concluding Remarks

The contributions to “Molecular Research in Asthma and Allergy” illustrate how modern allergy research is moving beyond a single-pathway account of disease. Immune regulation, epithelial signaling, gene–environment interactions, immunometabolic effects, allergen penetration, and increasingly granular diagnostic approaches are not isolated research directions; they describe different levels of one interconnected biological problem. The challenge ahead is to integrate these levels without overstating the clinical maturity of individual findings. Such an approach offers a credible foundation for more precise diagnosis, better patient stratification, and ultimately more individualized prevention and treatment.

As Guest Editor, I sincerely thank the contributing authors for their scientific work, the reviewers for their careful assessments, and the editorial team of the *International Journal of Molecular Sciences* for supporting this Special Issue. I hope these contributions stimulate further collaboration across molecular immunology, allergology, dermatology, pediatrics, and translational medicine.

## Data Availability

No new data were created or analyzed in this study. Data sharing is not applicable to this article.
